# Combined serum albumin, fecal immunochemical test, and leucine-rich alpha-2 glycoprotein levels for predicting prognosis in remitting patients with ulcerative colitis

**DOI:** 10.1038/s41598-023-41137-x

**Published:** 2023-08-24

**Authors:** Naohiro Nakamura, Yusuke Honzawa, Shuhei Nishimon, Yasuki Sano, Yutaro Tokutomi, Yuka Ito, Naoto Yagi, Sanshiro Kobayashi, Mamiko Aoi, Tomomitsu Tahara, Norimasa Fukata, Toshiro Fukui, Makoto Naganuma

**Affiliations:** Division of Gastroenterology and Hepatology, Third Department of Internal Medicine, Kansai Medical University, 2-5-1, Shinmachi, Hirakata, Osaka 573-1010 Japan

**Keywords:** Gastroenterology, Gastrointestinal diseases

## Abstract

This study investigated the usefulness of serum leucine-rich alpha-2 glycoprotein (LRG) and fecal immunochemical tests (FIT) for predicting relapse in patients with ulcerative colitis (UC). Data of 194 patients tested for LRG between January 2020 and June 2022 were retrospectively collected and clinical characteristics were recorded. LRG was strongly correlated with CRP levels and it had a moderately negative correlation with albumin levels, whereas FIT was not significantly correlated with either CRP or albumin levels. Furthermore, the median serum albumin and FIT were significantly different between patients with or without clinical relapse; while the LRG level was not associated with clinical relapse. Although LRG is not an independent factor for predicting clinical relapse, the cumulative remission rate was significantly higher in patients with higher albumin than in those with lower albumin. Furthermore, the combination of FIT and albumin was useful for predicting for relapse, patients with higher FIT and lower albumin tended to have higher relapse rates than those with both lower FIT and albumin and those with lower FIT and higher albumin. Our study indicated that serum albumin level is useful for predicting relapse, even in remitting outpatients. Although LRG is not an independent factor for predicting clinical relapse, it is useful for identifying patients that are likely to relapse when combined serum albumin or FIT results.

## Introduction

Ulcerative colitis (UC) is a chronic inflammatory disease of the colon, with increasing incidence in Japan over the last 50 years. UC is characterized by periods of remission and relapse, which can significantly reduce quality of life. Medical treatment, including biologics and Janus kinase (JAK) inhibitors, to maintain clinical remission is therefore important. Recently, the importance of endoscopic assessment has been emphasized considering that approximately one-third of patients in clinical remission exhibit signs of endoscopic inflammations^1^. Endoscopic remission has been associated with better prognosis and a low risk of hospitalization and future colectomy^2–4^. Given these associations, selecting therapeutic targets in inflammatory bowel disease (STRIDE-II) was published, in which the authors described endoscopic remission as a long-term target, and recommended therapy modification if endoscopic remission is not achieved. Endoscopy is useful for assessing the severity of mucosal inflammation and therapeutic response before and after treatment. Although endoscopic evaluation of the entire colon, including the terminal ileum, is the gold standard, it is limited by patient burden, surgeon burden, and cost, and should therefore not be performed frequently. Identification of biomarkers for non-invasive assessment of the severity of mucosal inflammation in inflammatory bowel disease (IBD) is therefore important.

Fecal calprotectin (FCP), which leaks from the intestinal mucosa into the lumen, has been identified as a non-invasive biomarker^5^ of endoscopic severity^6,7^, therapeutic response^8^, and long-term clinical prognosis of patients with UC^9,10^, and is currently widely used in clinical practice. However, the Japanese health insurance system only allows FCP determination once every three months. Furthermore, FCP has low disease specificity, and inflammation related to diverticulitis and colonic cancer may also elevate calprotectin levels.

The fecal immunochemical test (FIT) was originally designed to detect a small amount of intestinal bleeding using antibodies specific for hemoglobin in human red blood cells, which strongly correlates with the degree of colonic bleeding. In recent years, several studies have used the FIT to evaluate endoscopic activity in patients with UC, as FIT levels correlate with endoscopic severity, mucosal healing, and clinical prognosis^11,12^. Furthermore, the FIT is a simple, inexpensive method that can be repeated in an outpatient setting with immediate results and is therefore very useful in clinical practice. However, the FIT can yield false positive results due to hemorrhoids and inflammatory polyps without endoscopic mucosal inflammation. When they are used alone, they have some limited predictive ability. Previous studies have reported that a combination of FCP and FIT can identify remitting UC patients^13^.

Serum leucine-rich alpha-2 glycoprotein (LRG) was identified as a potential biomarker for inflammatory in patients with rheumatoid arthritis^14^. Serum LRG levels have been also reported to correlate with endoscopic severity^15^ and mucosal healing^16^ in patients with UC exhibiting endoscopic severity. Furthermore, serum LRG concentrations were significantly elevated in active UC patients compared with patients in remission, and compared to CRP, LRG was better correlated with disease activity in UC^17^. Although numerous studies have reported the usefulness of LRG for predicting endoscopic severity and clinical outcome of medical treatment, few studies have evaluated the relationship between LRG and long-term prognosis in patients with clinical remission. This study evaluated the usefulness of LRG in addition to albumin and FIT for predicting clinical recurrence in remitting patients with UC.

## Methods

### Study design and patients

In this single-center retrospective observational study, we analyzed the medical records of patients with UC who had LRG levels determined at the clinic of the Division of Gastroenterology and Hepatology, Kansai Medical University Hospital (Osaka, Japan), between January 2020 and June 2022. All patients with UC were diagnosed in accordance with the guidelines of the Research Committee on Inflammatory Bowel Disease in Japan. All methods of this study were carried out in accordance with relevant guidelines and regulations of Kansai Medical University, and it was approved by the ethics committees as a partial investigation of factors predicting IBD diagnosis and treatment efficacy (2022136). Informed consent was obtained from participants and/or their legal guardians while the ethics committees approved that patients were also allowed an opt-out approach to refuse study participation via the institutional website as the study posed no risk to participants.

### Study procedures

Patient demographic data was collected and disease characteristics, including sex, age, disease duration, disease extent, blood biomarkers (hemoglobin, platelet count, serum albumin, CRP level, and erythrocyte sedimentation rate), partial Mayo score, and medications at the time of study entry were recorded (Table 1). The partial Mayo score (range 0–9) was defined as the sum of three of four Mayo sub-scores for daily diarrhea, rectal bleeding, and physician’s assessment. The Mayo endoscopic sub-score was excluded.

**Table 1:** Clinical characteristics at study entry (n = 194).

Male/female	98/96
Age [years] median [range]	40.5 [13–88]
Clinical remission, n [%]	157 [80.9]
Duration of disease [years] median [range]	7 [0–35]
Observation [days] median period	336 [0–518]
Extent	
Total colitis, n [%]	107 [55.2]
Left-sided colitis, n [%]	58 [29.9]
Proctitis, n [%]	26 [13.4]
Right-sided or segmental colitis, n [%]	3 [1.5]
Clinical course	
Relapse-remittng, n [%]	142 [73.2]
Chronic continuous, n [%]	7 [3.6]
First attack, n [%]	45 [23.2]
Hemoglobin [g/dL] median [range]	13.7 [9.2–18.2]
CRP [mg/dL] median [range]	0.0455 [0.005–6.65]
Albumin [g/dL] median [range] (n = 192)	4.4 [2.6–5.1]
Platelet [× 10^4^ μL] median [range]	24.5 [12.4–51.1]
LRG [μg/mL] median [range]	10.9 [5–48.7]
FIT [ng/mL] median [range] (n = 128)	30.5 [20–173910]
pMayo score, median [range]	0 [0–8]
Concomitant medication	
5-ASA	
Oral, n [%]	166 [85.6]
Enema, n [%]	13 [6.7]
Suppository, n [%]	26 [13.4]
Steroids	
Oral, n [%] [range of daily dose]	12 [6.2] [2.5–30]
Rectal, n [%]	23 [11.9]
Thiopurine	
AZA, n [%] [range of daily dose]	29 [15.0] [20–100]
6-MP, n [%] [range of daily dose]	9 [9.6] [10–45]
Biologics	
Anti-TNF agent, n [%]	23 [11.9]
Anti-α4β7-integrin, n [%]	14 [7.2]
Anti-IL-12/23p40 antibody, n [%]	4 [2.1]
JAK inhibitor, n [%]	9 [4.6]
Tacrolimus, n [%]	1 [0.5]

*CRP* C-reactive protein, *LRG* leucine-rich alpha-2-glycoprotein, *FIT* fecal immunochemical test, *pMayo score* partial Mayo score, *5-ASA* 5-aminosalicylic acid, *AZA* azathioprine, *6-MP* 6-mercaptopurine, *TNF* tumor necrosis factor, *IL* Interleukin.

### Definition of remission and relapse

Clinical remission was defined as a partial Mayo score ≤ 2 points, with no individual sub-score exceeding 1 point. Clinical relapse was defined as a partial Mayo score ≥ 3 points or patients who required an addition or modification of medication for clinical recurrence.

### Assessment of fecal immunochemical blood test and LRG

Stool samples were collected from the patients and delivered to the hospital within three days. FIT levels were measured by enzyme-linked immunosorbent assay using an L-type IG auto Hem (FUJIFILM Wako Pure Chemical Corporation, Osaka, Japan). LRG levels were measured using Nanopia LRG^®^ (Sekisui Medical, Tokyo, Japan). The limit of detection for LRG, below 5 µg/mL, was defined as 5 µg/mL, while the limit of detection for FIT, below 20 ng/mL, was defined as 20 ng/mL.

### Outcomes and statistical analyses

Categorical variables are described as absolute numbers and relative frequencies using percentages, and continuous variables are described as medians and ranges. The primary endpoint was to assess the association between clinical characteristics, including LRG, at baseline, and clinical relapse in remitting patients with UC, using the log-rank test with Bonferroni correction and Cox proportional hazard model. The main secondary endpoints were to assess correlations between LRG, FIT, clinical severity (partial Mayo score), and serum CRP, elevated sedimentation rate (ESR), platelet, and serum albumin levels in patients with UC using the Spearman rank correlation test, Kruskal–Wallis test, and Steel–Dwass test. In addition, median age, disease duration, partial Mayo score, CRP, ESR, hemoglobin, platelet level, LRG, and FIT levels of patients in remission and relapse were compared using the Mann–Whitney U test. The proportion of sex and extent of disease in the remission and relapse groups were compared using the chi-square or Fisher’s exact test. To determine the cut-off value of LRG and FIT for the prediction of clinical relapse, receiver operating characteristic (ROC) curves were plotted, and the areas under the curve (AUC) were calculated to predict the diagnostic ability of LRG and FIT for clinical relapse. Statistical significance was set at P < 0.05. JMP Pro version 16.2.0 (SAS. Institute Inc., Cary, NC, USA) was used for all statistical analyses. As this study aimed to assess the usefulness of biomarkers for predicting long-term outcomes in a clinical real-world setting, we did not collect data regarding endoscopic severity because, in most cases, endoscopic procedures were not conducted simultaneously with LRG and FIT determinations.

### Ethics statement

The study was approved by the Ethics Committees of Kansai Medical University as a partial investigation of factors predicting IBD diagnosis and treatment efficacy (2022136). As the study posed no risk to participants, patients were allowed an opt-out approach to refuse study participation in this study via the institutional website.

## Results

### Clinical characteristics at study entry

**Figure 1:**
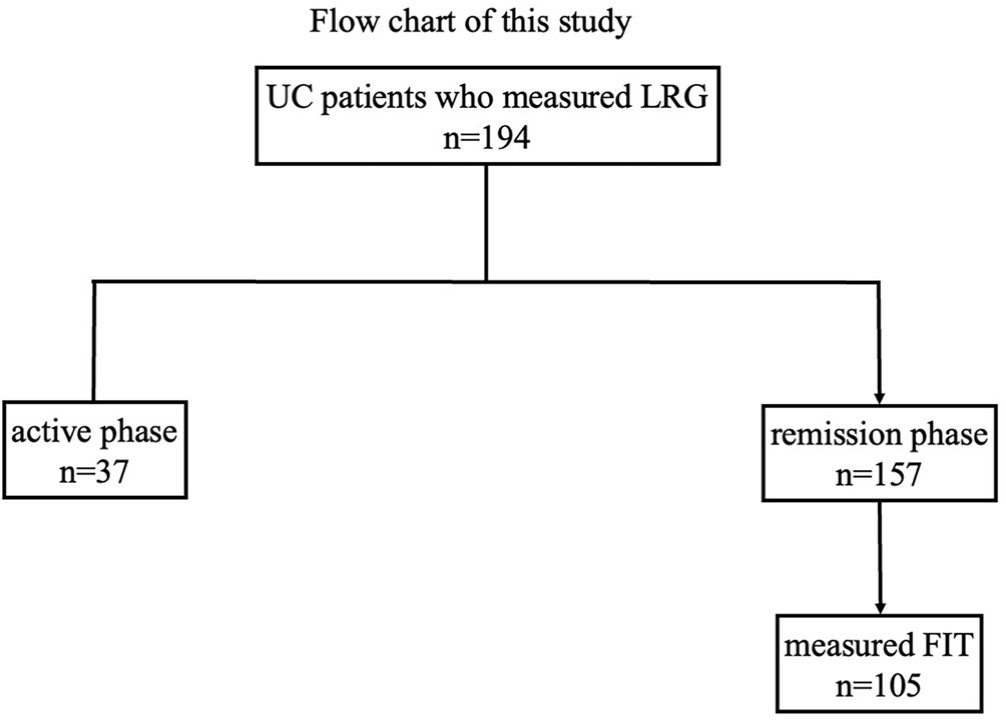
Flow chart of the study design. *UC* ulcerative colitis, *LRG* leucine-rich alpha-2-glycoprotein, *FIT* fecal immunochemical test.

The clinical characteristics of the 194 patients with UC are summarized in Table 1. Among these,157 patients (80.9%) were in clinical remission at study entry and 107 patients (55.2%) had extensive colitis (Fig. 1). The median CRP level, hemoglobin level, and platelet count were normal. The median FIT and LRG levels were 30.5 ng/mL and 10.9 μg/mL, respectively. Upon study entry, 85.6% of the patients were treated with oral 5-aminosalicylic acid (5-ASA), 19.6% were treated with thiopurine, and approximately 25% received advanced therapy, such as biologics or JAK inhibitor tofacitinib (Table 1).

### Correlation between FIT, LRG, and clinical severity

**Figure 2:**
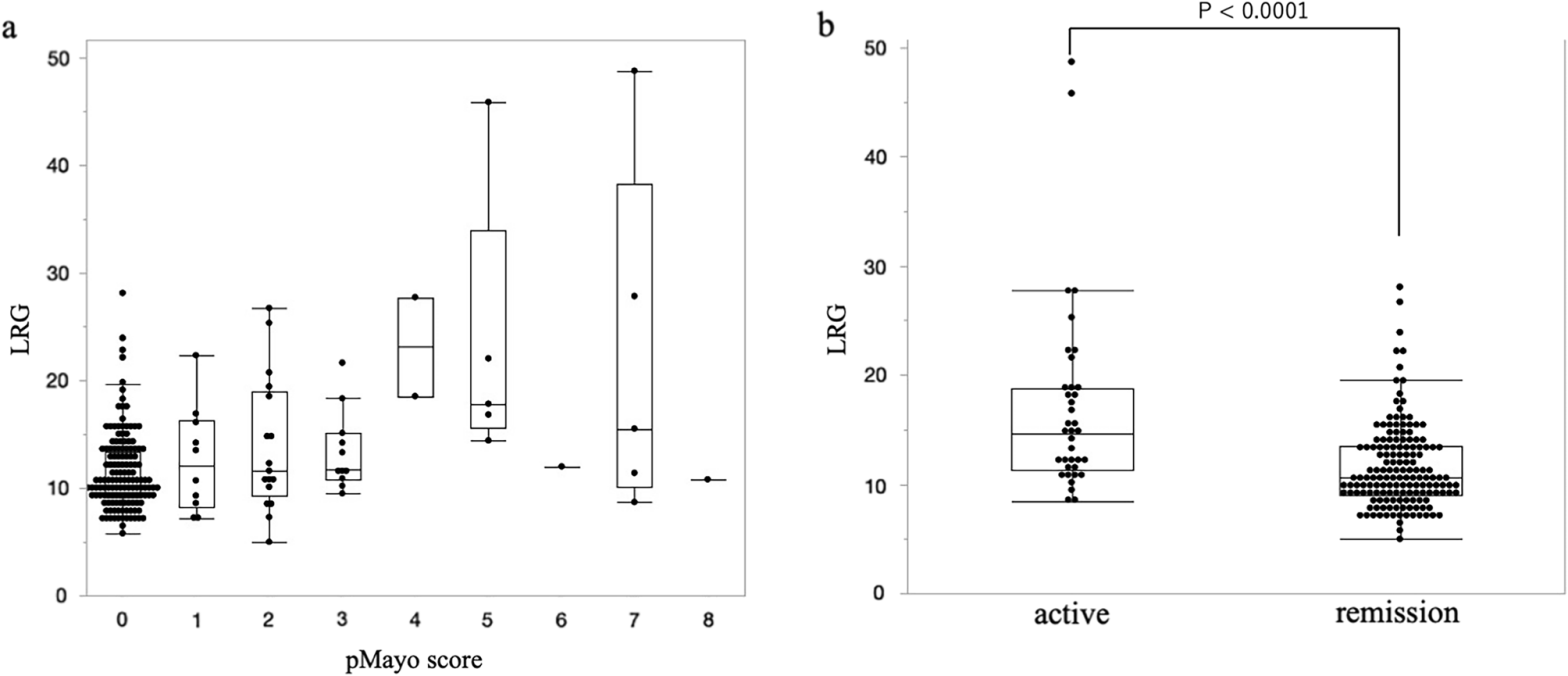
(**a**) Analysis of LRG levels in patients with different partial Mayo scores (P = 0.0054; Kruskal–Wallis test); the partial Mayo score correlates with a gradual increase in LRG level (r = 0.2707, P = 0.0001; Spearman's rank correlation coefficient). (**b**) Analysis of LRG levels in patients in active and remitting phases (P < 0.0001; Mann–Whitney U test). *LRG* leucine-rich alpha-2-glycoprotein, *pMayo score* partial Mayo score.

Evaluation of correlations between clinical severity, FIT, and serum biomarkers, including LRG indicated that the partial Mayo score weekly correlates with LRG level (r = 0.2707, P = 0.0001), and LRG levels in active phase UC are significantly higher than those in clinical remission (P < 0.0001; Fig. 2a,b).

### Correlation between FIT, LRG, and other blood markers

LRG was strongly correlated with CRP (r = 0.7433), moderately correlated with albumin (r = − 0.5732) and ESR (r = 0.5682), and weakly correlated with platelet and hemoglobin levels (Table 2). Compared with LRG, FIT levels did not correlate with CRP (r = 0.2395), ESR (r = 0.1152), or albumin (r = − 0.2103) levels. Although a previous study indicated that both LRG and FIT correlated with endoscopic severity^12,15,17^, in our cohort, LRG was not correlated with FIT levels (r = 0.1932) (Table 2).

**Table 2:** Correlations between biomarkers, clinical activity, and blood markers.

	FIT	CRP	ESR	Alb	Plt	Hb
LRG (n = 194) 95% CI	0.1932 (0.0204–0.3549)	0.7433 (0.6729–0.8004)	0.5682 (0.4421–0.6722)	− 0.5372 (− 0.6308 to − 0.4282)	0.3687 (0.2403–0.4844)	− 0.3850 (− 0.4988 to − 0.2581)
p value	0.0289	< 0.0001	< 0.0001	< 0.0001	< 0.0001	< 0.0001
FIT (n = 128) 95% CI		0.2395 (0.06886–0.3966)	0.1152 (− 0.08839–0.3096)	− 0.2103 (− 0.3709 to − 0.0374)	0.007192 (− 0.1666–0.1805)	− 0.01456 (− 0.1876–0.1594)
p value		0.0065	0.2663	0.0177	0.9358	0.8704
CRP (n = 194) 95% CI			0.4621 (0.3185–0.5849)	− 0.3035 (− 0.4267 to − 0.1691)	0.2362 (0.09858–0.3649)	− 0.1448 (− 0.2799 to − 0.00396)
p value			< 0.0001	< 0.0001	0.0009	0.0440
ESR (n = 136) 95% CI				− 0.4703 (− 0.5926 to − 0.3268)	0.2550 (0.09051–0.4059)	− 0.5262 (− 0.6380 to − 0.3926)
p value				< 0.0001	0.0027	< 0.0001
Alb (n = 192) 95% CI					− 0.4703 (− 0.5926 to − 0.3268)	0.2550 (0.09051–0.4059)
p value					< 0.0001	0.0027
Plt (n = 194) 95% CI						− 0.2934 (− 0.4171 to − 0.1592)
p value						< 0.001

*5-ASA* 5-aminosalicylic acid, *LRG* leucine-rich alpha-2-glycoprotein, *FIT* fecal immunochemical test, *CRP* C-reactive protein, *ESR* erythrocyte sedimentation rate, *Alb* albumin, *Plat* platelet count, *Hb* hemoglobin, *CI* confidence interval.

### Analysis of fecal and serum markers as predictive factors for relapse of remitting patients

In this cohort, 157 patients were in clinical remission. Clinical relapse occurred in 34 of 143 remitting patients (23.8%) with LRG < 16 μg/mL (which is the cut-off value of the Mayo endoscopic score ≤ 1 or ≥ 2), and in 5 of 14 remitting patients (35.7%) with LRG ≥ 16 μg/mL. There was no significant difference in relapse rates between the two groups (P = 0.2635; log-rank test). The ROC curve revealed a cut-off value of 10, resulting in a relatively low AUC (0.56595) (Supplementary Fig. 1a), and there was no significant difference in relapse rate of patients with LRG ≦ 10 μg/mL and patients with LRG > 10 μg/mL (P = 0.4588; log-rank test) (Supplementary Fig. 1b).

To investigate prognostic markers in remission, we analyzed 105 clinically remitting patients in whom both LRG and FIT were determined simultaneously. During the observation period (median of 336 days), 24 (22.9%) patients relapsed within a median duration of 196 days (range 14–451 days). Comparison of the clinical characteristics of the relapse group and remission group by univariate analysis indicated that median serum albumin, FIT levels, and partial Mayo score (1 vs 0) in patients with clinical relapse were significantly different with those in patients without clinical relapse (Table 3), while the median LRG levels of the two groups were comparable (10.5 μg/mL vs 11.7 μg/mL, P = 0.3106; Table 3). Multivariate analysis indicated that elevated serum albumin (P = 0.0220) and FIT (P = 0.0010) are independent predictors of clinical remission.

**Table 3:** Univariate and multivariate analyses for predicting clinical relapse in remitting patients.

	Non-relapse (n = 81)	Relapse (n = 24)	Univariate analysis	Multivariate analysis		
			p-value	HR	95% CI	p-value
Male/female	43/38	10/14	0.3908			
Age median [range]	40 [17–88]	48.5 [22–81]	0.2571			
Duration of disease [years] median [range]	7 [0.08–29]	8 [0.17–24]	0.7711			
LRG median [range]	10.5 [5–28.1]	11.7 [7.3–23.9]	0.3106			
Hemoglobin [g/dL] median [range]	14.1 [10.1–18.2]	13.4 [10.9–16.6]	0.4408			
CRP [mg/dL] median [range]	0.041 [0.008–0.868]	0.0355 [0.008–0.587]	0.3581			
ESR [mm/h] median [range] n = 74	5.2 [0.2–36.8] (n = 63)	6.3 [5.3–16.7] (n = 11)	0.8610			
Alb [g/dL] median [range] n = 104	4.5 [3.8–5.1] (n = 80)	4.3 [3.2–5.1] (n = 24)	0.0108	0.1868	0.0435–0.7705	0.0220
Platelet [× 10^4/^μL] median [range]	23.9 [12.4–51.1]	22.75 [15.2–37.1]	0.7278			
FIT median [range]	20 [20–24224]	48.5 [20–39596]	0.0161	1.0001	1.0000–1.0001	0.001
pMayo	0 [0–2]	0 [0–1]				
1/0			0.0315			
2/1			0.9989			

*LRG* leucine-rich alpha-2-glycoprotein, *CRP* C-reactive protein, *ESR* erythrocyte sedimentation rate, *Alb* albumin, *FIT* fecal immunochemical test, *pMayo score* partial Mayo score, *HR* hazard ratio, *CI* confidence interval.

**Figure 3:**
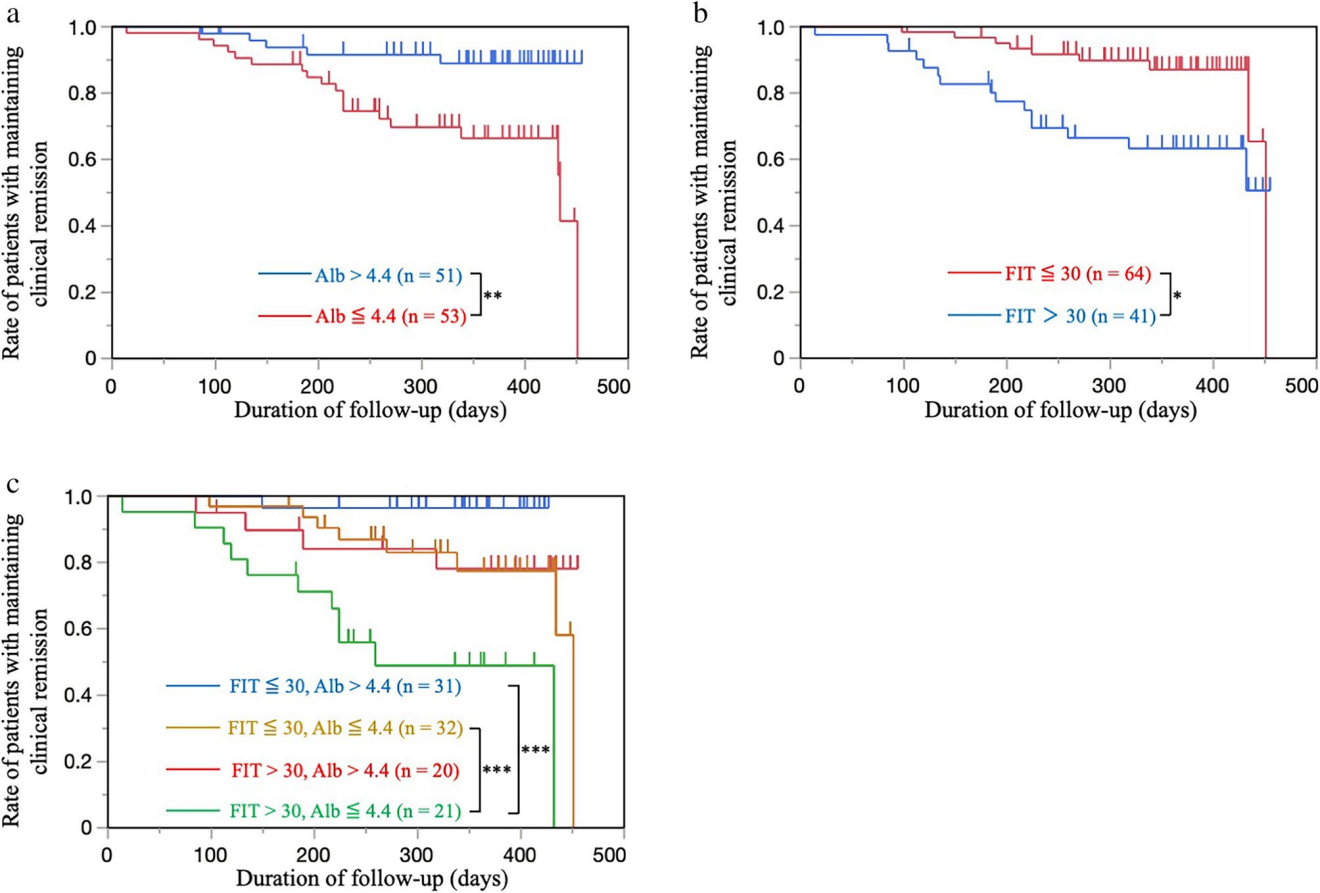
(**a**) Cumulative non-recurrence rates in patients with serum albumin ≤ 4.4 mg/mL and patients with albumin > 4.4 mg/mL (P = 0.0034; log-rank test). (**b**) Cumulative non-recurrence rates in patients with FIT ≤ 30 ng/mL and patients with FIT > 30 ng/mL (P = 0.0100; log-rank test). (**c**) Cumulative non-recurrence rates in patients with (1) FIT ≤ 30 ng/mL and Alb > 4.4 g/dL, (2) FIT ≤ 30 ng/mL and Alb ≤ 4.4 g/dL, (3) FIT > 30 ng/mL and Alb > 4.4 g/dL, and (4) FIT > 30 ng/mL and Alb ≤ 4.4 g/dL. Patients the patients with FIT > 30 ng/mL and Alb ≤ 4.4 g/dL have higher relapse rates than those with FIT ≤ 30 ng/mL and Alb ≤ 4.4 g/dL (P = 0.0041; log-rank test with Bonferroni correction) and those with FIT ≤ 30 ng/mL and Alb > 4.4 g/dL (P = 0.0001; log-rank test with Bonferroni correction). *P < 0.05, **P < 0.01, ***P < 0.0083. *LRG* leucine-rich alpha-2-glycoprotein, *FIT* fecal immunochemical test, *Alb* albumin.

Next, we determined the cut-off values of albumin and FIT levels to identify the clinical relapse risk group. The ROC curve revealed an AUC of 0.65417 for albumin, and at a cut-off value of 4.4 mg/mL, sensitivity and specificity were 0.7917 and 0.5750, respectively. The ROC curve revealed an AUC of 0.65226 for FIT, and at a cut-off value of 30 ng/mL, sensitivity was 0.6250 and specificity was 0.6667 for cumulative clinical relapse in remitting patients with UC (Supplementary Fig. 2). The cumulative remission rate was significantly higher in patients with albumin > 4.4 g/dL than in patients with albumin ≤ 4.4 g/dL (P = 0.0034; Fig. 3a). The cumulative remission rate was also higher in patients with FIT ≤ 30 ng/mL than in patients with FIT > 30 ng/mL (P = 0.0100; Fig. 3b). The patients with FIT > 30 ng/mL and Alb ≤ 4.4 g/dL have higher relapse rates than those with FIT ≤ 30 ng/mL and Alb ≤ 4.4 g/dL (P = 0.0041; log-rank test with Bonferroni correction) and those with FIT ≤ 30 ng/mL and Alb > 4.4 g/dL (P = 0.0001; log-rank test with Bonferroni correction, Fig. 3c).

### Medical treatment at the time of relapse

Relapse, characterized by worsening of symptoms or endoscopic findings, occurred in 24 patients during the median follow-up period of 196 days (range 14–451 days). Detailed data regarding medication at the time of relapse are shown in Supplementary Table 1.

## Discussion

Treat-to-target strategies are widely used in the management of patients with IBD. STRIDE, initiated in 2015 using an evidence-based expert consensus process^18^, introduced the term “treat-to-target” and proposed patient-reported outcome and endoscopic remission as targets in IBD treatment. The 2021 updated STRIDE-II incorporated time-dependent treatment targets to facilitate IBD treatment and improve clinical outcomes^19^. Candidates for UC treatment targets included endoscopic improvement, serum and fecal biomarker normalization, and clinical remission. Although endoscopic remission is associated with improved prognosis in patients with UC^20^, confirming endoscopic remission is invasive, and colonoscopies should not be performed frequently. Identification of non-invasive biomarkers to assess the severity of mucosal inflammation is therefore necessary. We previously reported the usefulness of FCP and FIT in predicting endoscopic severity and long-term prognosis in patients with UC, demonstrating that the combination of FCP and FIT increases diagnostic accuracy compared with either FCP or FIT^13^.

Previous studies also reported LRG as a useful biomarker for endoscopic severity of IBD^15,16,21–24^ and for assessing disease activity in IBD patients receiving adalimumab^25^. However, few studies have investigated the usefulness of LRG as a biomarker to predict clinical recurrence in remitting patients. In this study, we therefore evaluated the usefulness of LRG, as well as LRG combined with a fecal biomarker, for prediction of clinical relapse in remitting patients. Since simultaneous measurement of both LRG and FCP is not allowed due to the regulations of the Japanese health insurance system, few clinical data of both LRG and FCP are available. We therefore assessed the usefulness of combined LRG and FIT for predicting clinical relapse. We found that although LRG was moderately correlated with blood markers (such as CRP and ESR) and clinical activity, it is not an independent predictor of clinical remission. In a clinical setting, LRG may therefore be more useful in assessing disease activity at the time of measurement than in predicting clinical relapse.

Furthermore, we confirmed that serum albumin level is an independent predictive factor for relapse in remitting patients. Previous studies have reported that serum albumin levels may predict disease severity and clinical outcome^26–30^. We found a decrease in serum albumin levels correlated to serum CRP and ESR, which may be attributed to albumin leaking from the intestinal mucosa and deteriorating nutritional status due to poor eating. Previous studies have also reported that low serum albumin levels are a significant predictor of colectomy^26–29^, and disease activity at diagnosis combined with low hemoglobin and serum albumin levels after initial therapy induction are significant predictors of relapse and colectomy^27,30^. However, these studies examined the relationship between albumin levels and intestinal resection in severe cases. Our results indicated that the cumulative clinical remission rate was higher in patients with albumin > 4.4 g/dL than those with albumin ≤ 4.4 g/dL, suggesting that serum albumin levels may help identify patients prone to relapse. Within the treat-to-target framework, patients with lower serum albumin levels may therefore benefit from a colonoscopy to assess the severity of mucosal inflammation. Furthermore, our results indicated that combining FIT and albumin may allow identification of patients that are likely to experience clinical relapse. We found that patients with FIT > 30 ng/mL and Alb ≤ 4.4 g/dL exhibit higher relapse rates, suggesting that careful follow-up, including FCP and colonoscopy, is necessary in these patients.

This study had some limitations. First, the sample size was not calculated to assess the predictive factors for clinical relapse. Second, we did not collect data regarding endoscopic severity. As our study examined whether serum markers and LRG in routine medical care contribute to long-term prognosis, colonoscopies were only conducted simultaneously with FIT and LRG measurement in approximately 17% (33/194) of the patients (data not shown). Third, FIT and LRG are not specific markers for IBD; therefore, we were unable to exclude hemorrhoidal bleeding because colonoscopy was not performed in all cases. Finally, we did not investigate the usefulness of combined LRG and FCP to predict relapse. However, previous studies have reported comparable diagnostic abilities of FCP and FIT, which suggests that the results of combined LRG and FCP may be comparable to the results of combined LRG and FIT^13^.

In conclusion, our study highlighted the advantages and limitations of FIT and albumin in monitoring patients with UC. The LRG levels were significantly higher in the clinically active phase than in the remission phase. We found that serum albumin level is a predictive factor for clinical relapse and FIT could identify patients who are at risk of relapse. Although LRG is not an independent factor for predicting clinical relapse, it is useful for identifying patients that are unlikely to relapse when combined serum albumin or FIT results.

### Supplementary Information

Supplementary Information.

## Data Availability

The raw data for this study was shared with all the authors. The datasets generated and/or analyzed during the current study are not publicly available considering that we did not receive the permission that data is currently accessible to researchers from outside organizations at present. However, it will be available from the corresponding author on reasonable request after permission will be obtained by the ethnic committee of Kansai Medical University.
