# Supplementary material for: Combined serum albumin, fecal immunochemical test, and leucine-rich alpha-2 glycoprotein levels for predicting prognosis in remitting patients with ulcerative colitis
Source: Sci Rep. 2023 Aug 24;13:13863. doi: 10.1038/s41598-023-41137-x (PMC10449766; doi:10.1038/s41598-023-41137-x)

**Supplemental Table.** Medical treatment at clinical relapse in remitting patients in whom both LRG and FIT were measured.

| Initiation of 5-ASA formulation | 10 [41.7] |
| --- | --- |
| oral, n [%] | 1 |
| suppository, n [%] | 7 |
| enema, n [%] | 2 |
| Switch to other class of 5-ASA | 4 [16.7] |
| Increase dose of 5-ASA, n [%] | 2 [8.3] |
| Budesonide rectal foam, n [%] | 7 [29.2] |
| Thiopurine, n [%] | 1 [4.2] |
| Anti-TNF agent, n [%] | 1 [4.2] |
| JAK inhibitor, n [%] | 2 [8.3] |

LRG, leucine-rich alpha-2-glycoprotein; FIT, fecal immunochemical test; 5-ASA, 5-aminosalicylic acid; LRG, leucine-rich alpha-2-glycoprotein; FIT, fecal immunochemical test; TNF, tumor necrosis factor.

**Supplemental Figure 1.** Analysis of patients in remission (n = 157).

(a) ROC curves and the AUC for prediction of the diagnostic ability of LRG in remitting patients. (b) Cumulative non-recurrence rates in patients with LRG ≤ 10 μg/mL and patients with LRG > 10 μg/mL (P = 0.4588; log-rank test).

AUC, area under the curve; LRG, leucine-rich alpha-2-glycoprotein; ROC, receiver operating characteristic.


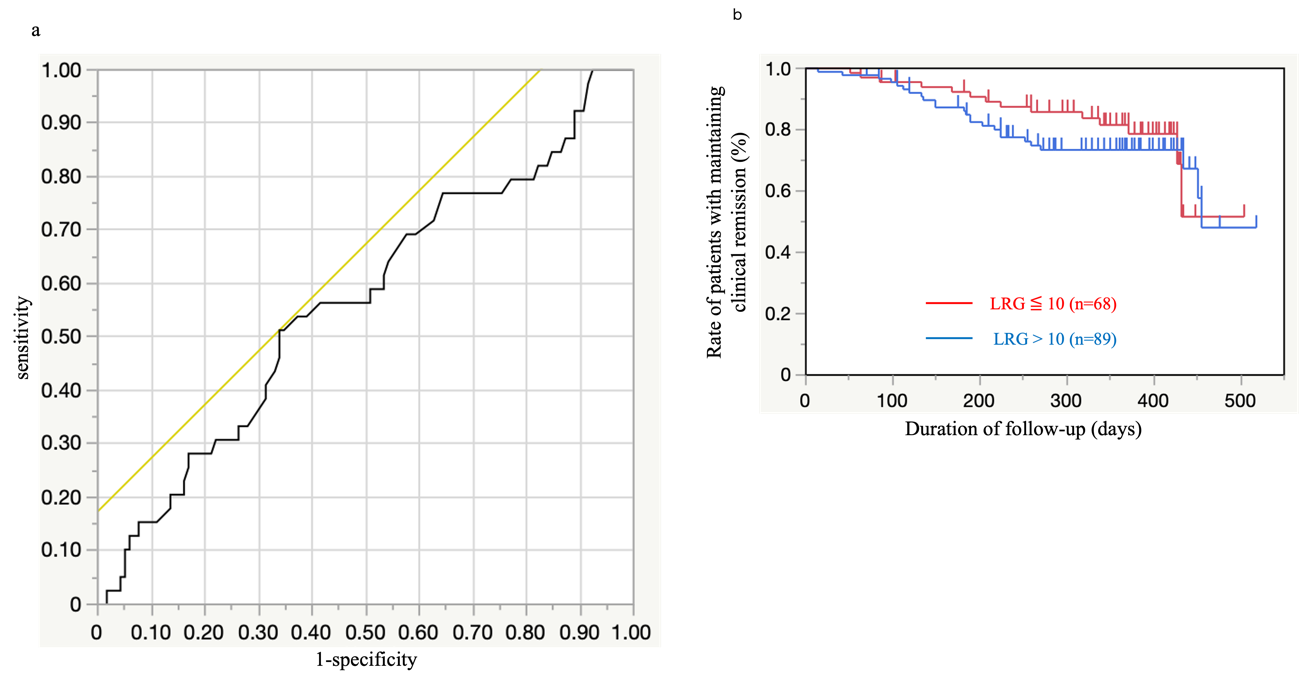


**Supplemental Figure 2.** Analysis of patients in remission (n = 105).

ROC curves and the AUC for prediction of the diagnostic ability of FIT in remitting patients.

AUC, area under the curve; FIT, fecal immunochemical test; ROC, receiver operating characteristic.


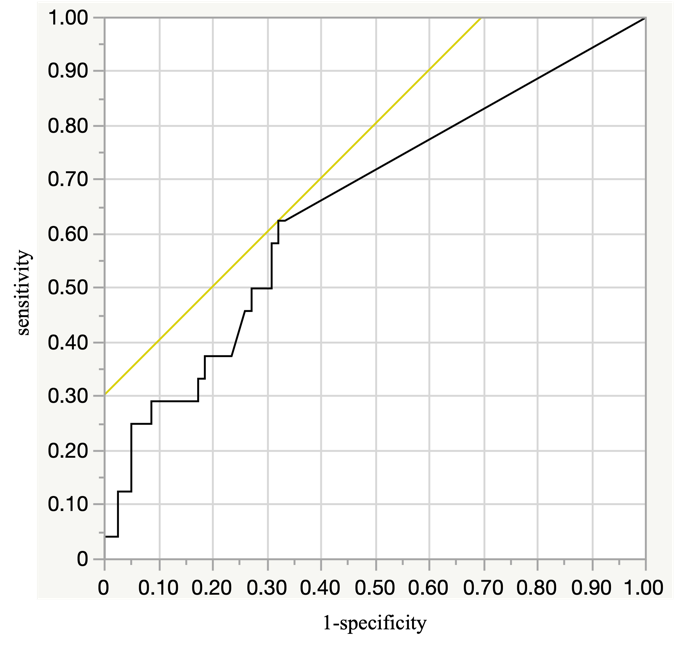

Supplement: Supplementary file 1 — Supplementary Information. [file 41598_2023_41137_MOESM1_ESM.docx]
